# Effect of chromium supplementation on glycated hemoglobin and fasting plasma glucose in patients with diabetes mellitus

**DOI:** 10.1186/1475-2891-14-14

**Published:** 2015-02-13

**Authors:** Raynold V Yin, Olivia J Phung

**Affiliations:** Western University of Health Sciences, College of Pharmacy, 309 E. Second St, Pomona, CA 91766 USA

**Keywords:** Chromium, Diabetes mellitus, Fasting plasma glucose, Glycated hemoglobin, Meta-analysis, Systematic review

## Abstract

**Aims:**

Chromium (Cr) is a trace element involved in glucose homeostasis. We aim to evaluate and quantify the effects of Cr supplementation on A1C and FPG in patients with T2DM.

**Materials and methods:**

A systematic literature search of Pubmed, EMBASE and the Cochrane Library (from database inception to 11/2014) with no language restrictions sought RCTs or cohort studies evaluating Cr supplementation in T2DM vs control and reporting either change in glycated hemoglobin (A1C) or fasting plasma glucose (FPG). Meta-analysis was conducted on each subtype of Cr supplement separately, and was analyzed by random effects model to yield the weighted mean differences (WMD) and 95% confidence intervals (CIs). Heterogeneity was assessed by using the I^2^ statistic.

**Results:**

A total of 14 RCTs (n = 875 participants, mean age range: 30 to 83 years old, 8 to 24 weeks of follow-up) were identified (Cr chloride: n = 3 study, Cr picolinate: n = 5 study, brewer’s yeast: n = 4 study and Cr yeast: n = 3 study). Compared with placebo, Cr yeast, brewer’s yeast and Cr picolinate did not show statistically significant effects on A1C. Furthermore, compared to control, Cr chloride, Cr yeast and Cr picolinate showed no effect on FPG, however, brewer’s yeast showed a statistically significant decrease in FPG -19.23 mg/dL (95% CI = -35.30 to -3.16, I^2^ = 21%, n = 137).

**Conclusions:**

Cr supplementation with brewer’s yeast may provide marginal benefits in lowering FPG in patients with T2DM compared to placebo however it did not have any effect on A1C.

## Introduction

Type 2 diabetes mellitus (T2DM) is the most prevalent form of diabetes worldwide [1, 2]. It is characterized by defects in pancreatic insulin secretion or action causing hyperglycemia attributable to disturbances in carbohydrate, fat and lipid metabolism [1]. The worldwide prevalence of T2DM is increasing and more than 366 million people are expected to be affected by the year 2030 [2]. Symptomatic adverse drug events associated with current therapy may affect the patients’ adherence and the overall risk-benefit ratio of a drug [3]. Despite the advances in modern medicine, T2DM continues to be a public health concern. In a study conducted by the Medical Expenditure Panel Survey, individuals with diabetes are 1.6 times more likely to use alternative medicine, such as chromium, as compared to individuals without [4, 5].

Chromium (Cr) is a common supplement used by many patients with T2DM for the purpose of improving glucose regulation and in 2002, sales of chromium supplements were estimated at $85 million [6]. According to the National Institute of Health: Office of Dietary Supplements, an adequate intake of Cr for men and women is 35 and 25 μg/day, resepectively [7, 8]. Chromium chloride is the naturally occurring trivalent variety of chromium found in common food sources such as: whole grains, broccoli, mushrooms and green beans. In contrast, Cr picolinate is the synthetic sibling of Cr chloride. Additional forms of Cr supplementation may also come from Cr yeast and brewer’s yeast. Chromium is an essential micronutrient linked to the regulation of many processes in the human body including glucose homeostasis. Chromium helps to regulate glucose homeostasis by activating insulin receptors through the oligopeptide chromudulin thereby increasing insulin signal transduction and sensitivity [8]. A deficiency in Cr may result in glucose intolerance, elevated circulating insulin, fasting hyperglycemia, and even impair growth [9].

In recent years, using Cr for treatment of T2DM has been called into question because of mixed results from published data. There is currently insufficient evidence in the literature to make a definitive conclusion about the effects of Cr on glucose control. Current trials evaluating Cr supplementation on glucose control has resulted in conflicting results. The aim of this systematic review and meta-analysis is to assess the effectiveness of Cr supplementation in lowering A1C and FPG in patients with T2DM.

## Materials and methods

### Data source and search strategy

A comprehensive systematic literature search of Pubmed, Embase, and the Cochrane Library was conducted from the inception of each database to November 2014 with no language restrictions applied. The search strategy combined keywords and medical subject headings related to Cr supplementation and T2DM. A manual search of references from relevant articles was also performed to identify any additional studies. For further details about the search strategy, see Appendix.

### Study selection, data abstraction and validity assessment

Studies were included in the analysis if they were either RCTs or observational trials in patients with T2DM evaluating Cr supplementation in any dose or form, and also reported any of the following endpoints: A1C or FPG. Two investigators were responsible for completing study selection, data abstraction and validity assessment for all trials evaluated in this study. Data abstraction was completed by each investigator independently using a standardized data abstraction tool. Each investigator was responsible for obtaining the following information from each trial: author, publication year, funding source, description of study population, inclusion and exclusion criteria, study design, duration of study and intervention details. Validity assessment of RCTs was completed using the Cochrane Risk of Bias Tool [10]. The validity assessment had six domains covering: randomization, allocation concealment, blinding, blinding of outcomes assessment, incomplete data reporting and selective reporting. Each domain was scored as either having a low, unclear, or high risk of bias. Any discrepancies were resolved through discussion.

### Data synthesis and statistical analysis

To explore and quantify the changes Cr supplementation has on A1C and FPG, each parameter was treated as a continuous variable. The weighted mean differences (WMDs) and accompanying 95% confidence intervals (CI) were pooled using a DerSimonian and Laird random-effects model [11]. Meta-analysis was conducted on each subtype of Cr supplement separately using StatsDirect software version 2.7.9. A p-value of <0.05 was considered statistically significant for all analyses. Moreover, statistical heterogeneity between individual trials was determined using the I^2^ statistic. Heterogeneity value ranges from 0 to 100, with 25, 50 and 75% representing low, moderate, and high risk of statistical heterogeneity, respectively. Publication bias was planned using visual inspection of funnel plots to identify the relationship between effect size and sample size and Egger’s weighted regression statistic was used to evaluate asymmetry [12, 13]; however, the small number of RCTs per analysis precluded the ability to statistically analyze this.

## Results

### Study characteristics

**Figure 1:**
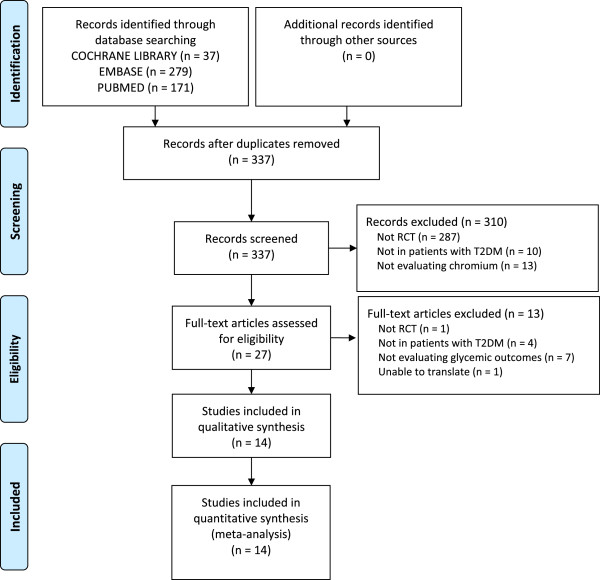
**PRISMA diagram.** Flowchart depicting when studies were excluded from the study and why.

**Figure 2:**
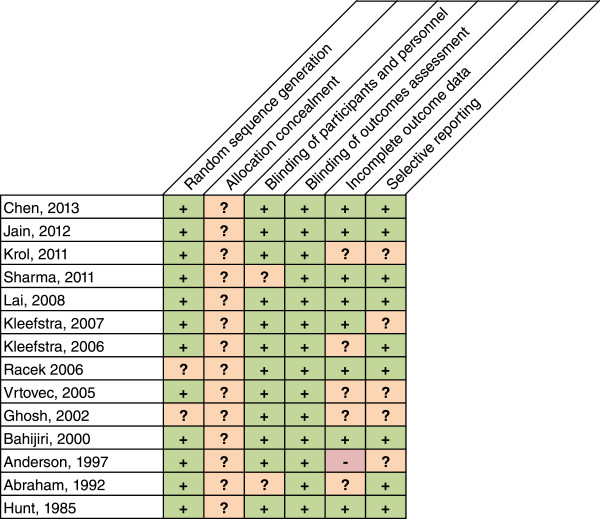
**Cochrane risk of bias assessment tool.** Six domains are evaluated, random sequence generation, allocation concealment, blinding of participants and personnel, blind of outcomes assessment, incomplete outcome data and selective reporting. – , ? and + represents either a high, unknown or low risk of bias, respectively.

Upon screening for inclusion and exclusion of studies, 14 nonduplicate RCTs (n = 875 patients, mean age range: 30 to 83 years old, 8 to 24 weeks of follow-up) met inclusion criteria (Figure 1) [13–27]. No observational studies were found to be eligible for inclusion in this study. Of the 14 unique studies identified, 11 reported results for A1C and 12 reported results for FPG. Patients in the intervention group received Cr supplement (dosing range 126 mcg-1,000 mcg daily) in the form of either: Cr chloride (n = 3) [17, 18, 20], Cr picolinate (n = 5) [16, 21, 23, 24, 28], Cr yeast (n = 3) [26, 27, 29], or brewer’s yeast (n = 4) [15, 18, 22, 25]. Cr was dosed one to three times daily (Table 1). Assessment for the Cochrane Risk of Bias tool is presented in Figure 2. The Cr levels for patients included in this meta-analysis were either not reported or within normal limits.

**Table 1:** **Baseline study characteristics**

Author, year, N	Inclusion criteria	Follow up	Product evaluated	Dose and frequency	Age (years), males (%) (mean ± SD)	Duration of DM (years) (mean ± SD)	Baseline A1C (%), FBG (mg/dL) (mean ± SD)	Baseline weight (kg), BMI (kg/m ^2^ ) (mean ± SD)
Chen, 2013, N = 66	30-75YO, T2DM ≥4 months, stable on OHA x3 months, FBG 140-250 mg/dL, A1C 7.5-12%, BMI 20-35 kg/m^2^	16 weeks	Chromium chloride (n = 38)	200 mcg, BID	62 ± 53, 58	NR	8.4 ± 8.6, 171 ± 314	NR, 28 ± 26
			Placebo (n = 28)	---	54 ± 45, 75	NR	8.5 ± 6.88, 191 ± 389	NR, 26 ± 21
Jain, 2012, N = 50	T2DM, 30-55YO, signed consent, willingness to participate, not pregnant	3 months	Chromium picolinate (n = 25)	400 mcg, unspecified	51 ± 10, 28	NR	7.5 ± 1.6, 129 ± 44	104 ± 32, 37 ± 11
			Placebo (n = 25)	---	49 ± 10, 8	NR	7.84 ± 1.7, 142 ± 62	100 ± 28, 38 ± 9
Król, 2011, N = 40	T2DM, not pregnant or breastfeeding, without vitamin-mineral supplementation in the last 3 months	8 weeks	Brewer’s yeast (n = 20)	100 mcg, 2 tablets QAM, 2 tablets in the afternoon, 1 tablet QPM	55 ± 9 years old for N = 40, 55 for N = 50	11.5 ± 7.8 for N = 40	8.09 ± 2.17, 194 ± 61	NR, 35 ± 9
			Placebo (n = 20)	---	55 ± 9 years old for N = 40, 55 for N = 50	11.5 ± 7.8 for N = 40	7.95 ± 80, 167 ± 47	NR, 36 ± 10
Sharma, 2011, N = 40	Not specified, however, authors noted that patients who enrolled had T2DM	3 months	Brewer’s yeast (n = 20)	42 mcg, 3 capsules TID	35-67 years old for N = 40, NR	NR	9.5 ± 1.2, 198 ± 30	NR, 25 ± 9
			Placebo (n = 20)	---	35-67 years old for N = 40, NR	NR	9.30 ± 0.98, 226 ± 82	NR, 26 ± 4
Lai, 2008, N = 20	<56YO, DM × 5 years, FBG > 153 mg/dL, A1C > 8.5%	6 months	Chromium yeast (n = 10)	1,000 mcg, unspecified	53 ± 2, 40	NR	10.2 ± 0.5, 225 ± 9	NR, 26 ± 0.9
			Placebo (n = 10)	---	51 ± 2, 50	NR	10.1 ± 0.4, 221 ± 17	NR, 26 ± 0.8
Kleefstra, 2007, N = 57	A1C 7–8.5%, only on OHG, no change to TX in 3 months, CrCl >50 mL/min, ALT <90 units/L	6 months	Chromium yeast (n = 29)	100 mcg, 2 tablets BID	68 ± 8, 62	6	6.9 ± 0.67, 157 ± 41	88 ± 20, 30 ± 6
			Placebo (n = 28)	---	66 ± 9, 61	4.5	7.01 ± 0.50, 144 ± 32.4	87 ± 17, 30 ± 6
Kleefstra, 2006, N = 46	<75YO, A1C >8%, daily insulin ≥50 units, CrCl >50 mL/min	6 months	Chromium picolinate (n = 14)	250 mcg, BID	60 ± 9, 29	13 ± 5	9.43 ± 1.09, NR	NR, 35 ± 7
			Chromium picolinate (n = 15)	500 mcg, BID	59 ± 6, 33	11 ± 6	9.67 ± 0.91, NR	NR, 33 ± 4
			Placebo (n = 17)	---	62 ± 8, 59	18 ± 8	9.41 ± 1.01, NR	NR, 34 ± 4
Racek, 2006, N = 36	>18YO, T2DM, agreement with study protocol	12 weeks	Chromium enriched yeast (n = 19)	100 mcg, 2 tablets BID	60.8 [37–80], 37	3	7.18 ± 1.52, 148 ± 46	NR, 34 ± 6
			Placebo (n = 17)	---	62 [47–77], 12	6	6.94 ± 1.68, 141 ± 43	NR, 35 ± 9
Vrtovec, 2005, N = 60	T2DM, without renal/hepatic dysfunction, without CAD/CVD	3 months	Chromium picolinate (n = 30)	1,000 mcg, unspecified	NR, NR	NR	6.9 ± 1, 160 ± 36	NR, 30 ± 4
			Placebo (n = 30)	---	NR, NR	NR	7.0 ± 1.5, 164 ± 43	NR, 31 ± 5
Ghosh, 2002, N = 100	T2DM, glycemic control ×3 months, not pregnant, no allergies to chromium picolinate, no multi-mineral supplementation	12 weeks	Chromium picolinate (n = 50)	200 mcg, BID	NR, NR	NR	7.2 ± 2.5, 124 ± 45	NR, NR
			Placebo (n = 50)	---	NR, NR	NR	7.2 ± 1.9, 122 ± 49	NR, NR
Bahijiri, 2000, N = 141	T2DM, negative history of pituitary, thyroid, kidney, or liver disease, no digestive problems, no vitamin supplements	8 months	Chromium chloride (n = 67)	200 mcg, unspecified	36-68 years old for N = 141, 38 for N = 141	NR	NR, NR	NR, 31 ± 8
			Brewer’s yeast (n = 74)	23.2 mcg, unspecified				
			Placebo (n = 69)	---				
Anderson, 1997, N = 155	Free of other disease, T2DM, 25-65YO, FBG between 130 mg/dL – 230 mg/dL, 2HOGTT between 170 mg/dL – 300 mg/dL, A1C between 8% – 12%	4 months	Chromium picolinate (n = 53)	100 mcg, BID	56 ± 1, NR	8 ± 1	9.4 ± 2.18, 184 ± 39	69 ± 2, 25 ± 0.5
			Chromium picolinate (n = 52)	500 mcg, BID	55 ± 1, NR	5 ± 0.7	9.4 ± 2.16, 176 ± 39	68 ± 1, 25 ± 0.4
			Placebo (n = 50)	---	56 ± 1, NR	5 ± 0.7	9.1 ± 2.12, 176 ± 38	69 ± 1, 25 ± 0.5
Abraham, 1992, N = 25	Authors did not clearly specify, however, they did note that patients with T2DM were enrolled and had established atherosclerotic disease	16 months	Chromium chloride (n = 13)	250 mcg, unspecified	NR, NR	NR	NR, 175 ± 36	NR, NR
			Placebo (n = 12)	---	NR, NR	NR	NR, 176 ± 42	NR, NR
Hunt, 1985, N = 39	T2DM, BG >140 mg/dL, A1C >8%	90 days	Brewer’s yeast (n = 22)	68 mcg/day, BID	62 ± 16 years old for N = 39, 73 for N = 79	NR	11.1, 204	123 ± 22 for N = 39, NR
			Placebo – Torula yeast (n = 17)	---	62 ± 16 years old for N = 39, 73 for N = 9	NR	11, 186	123 ± 22 for N = 39, NR

*Abbreviations: T2DM* type 2 diabetes mellitus, *OHA* oral hyperglycemic agent, *FBG* fasting blood glucose, *BID* twice daily, *TID* three times daily, *QAM* daily in the morning, *QPM* daily in the evening, *DM* diabetes mellitus, *TX* treatment, *CAD* coronary artery disease, *CVD* cardiovascular disease.

### Quantitative data synthesis

For each Cr subtype evaluated, publication bias was either not detected or unobtainable due to few studies.

### Cr chloride

**Figure 3:**
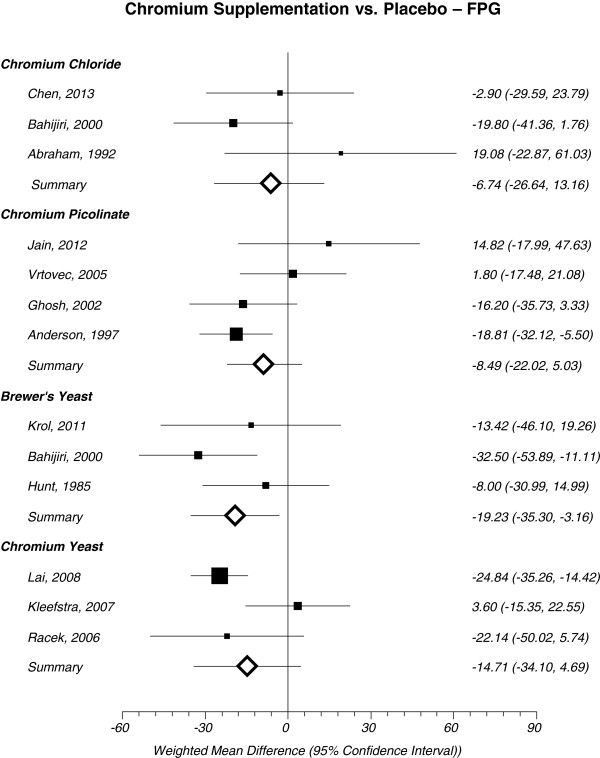
**Forest plot depicting results from each subtype of Cr supplement evaluating the effects of FPG vs. placebo based on year of publication.**

Chromium chloride is the naturally occurring trivalent variety of chromium found in common food sources such as: whole grains, broccoli, mushrooms and green beans. Results from the meta-analysis suggests that Cr chloride did not have a statistically significant effect on lowering FPG in patients with T2DM when compared to placebo -6.74 mg/dL (95% CI = -26.64 to 13.16, I^2^ = 31%) (Figure 3). Meta-analysis of Cr chloride on A1C could not be conducted because only one study reported this outcome [20].

### Cr picolinate

**Figure 4:**
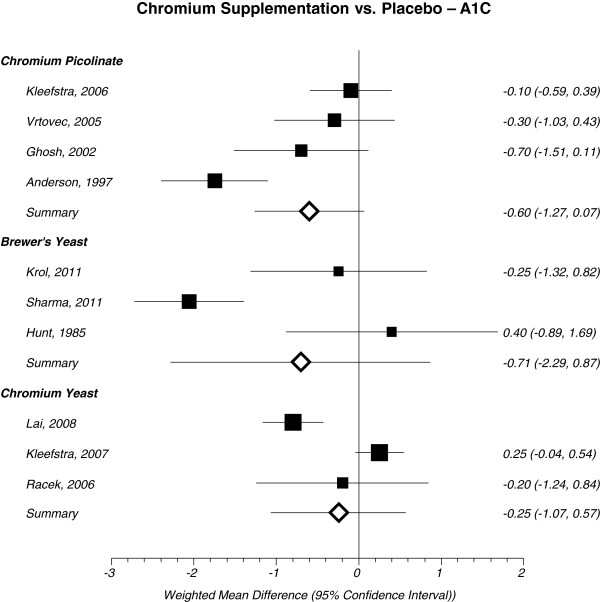
**Forest plot depicting results from each subtype of Cr supplement evaluating the effects of A1C vs. placebo based on year of publication.** Note: The squares represent the pooled results of that study in addition to all studies preceding it. Error bars represent 95% CIs and the diamond represents the overall pooled results. The solid vertical line extending upward from 0 is the null value.

Cr picolinate is the synthetic salt form of Cr chloride. Picolinic acid may serve to improve chromium absorption. Upon meta-analysis, Cr picolinate did not show a statistically significant improvement for lowering A1C -0.60% (95% CI = -1.27 to 0.07, I^2^ = 78%, n = 392) (Figure 4) or FPG -8.49 mg/dL (95% CI = -22.02 to 5.03, I^2^ = 48%) (Figure 3) in patients with T2DM.

### Cr yeast

An additional form of Cr supplementation may also come from Cr yeast. Results from this meta-analysis suggests that Cr yeast did not have a statistically significant improvement in lowering A1C 0.25% (95% CI = -1.07 to 0.57, I^2^ = 90%, n = 113) (Figure 4) or FPG -14.71 mg/dL (95% CI = -34.10 to 4.69, I^2^ = 70%) (Figure 3).

### Brewer’s yeast

Trivalent Cr is considered the biologically active form of Cr and it was originally discovered in brewer’s yeast [30]. Brewer’s yeast did not have a statistically significant effect on lowering A1C in patients with T2D -0.71% (95% CI = -2.29 to 0.87, I^2^ = 87%, n = 99) (Figure 4). However, brewer’s yeast demonstrated a statistically significant decrease in FPG -19.23 mg/dL (95% CI = -35.30 to -3.16, I^2^ = 21%) (Figure 3) in patients with T2DM compared to placebo.

## Discussion

Results from this study suggest that Cr does not have a statistically significant effect on A1C when administered in patients with T2DM. Furthermore, Cr chloride, Cr yeast or Cr picolinate did not improve FPG in patients with T2DM. However, brewer’s yeast had a statistically significant reduction in FPG when compared to placebo.

The limitations of this meta-analysis should be considered when interpreting these results. The high level of heterogeneity may be a limiting factor of this study. Visual inspection of funnel plots indicated the potential for publication bias and a lack of symmetry to some extent. Variables such as: age, gender, frequency of dose, formulation and duration of the study may have contributed to the high heterogeneity of the study. Although publication bias could not be statistically assessed; the comprehensive search strategy employed may have prevented the exclusion of relevant evidence. Furthermore, the trials included in this meta-analysis only included published trials.

Many factors may have contributed to the polar results obtained between Abraham et al. [17] and Bahijiri et al. [19] when exploring the effects of Cr chloride on FPG when compared to placebo (Figure 3). Changes in the standard of care for patients with T2DM between 1992 when Abraham et al. [17] was published and 2000 when Bahijiri et al. [19] was published may have contributed to the inconsistent results between the two studies. Furthermore, the patient population studied in Abraham et al. [17] recruited patients who were at high risk (i.e. previous myocardial infarction, moderate to severe intermittent claudication) compared to patients recruited by Bahijiri et al. [19]. Furthermore, Bahijiri et al. [19] only recruited patients who were either of Saudi origin or Arabs compared to Abraham et al. [17] who did not exclusively specify ethnic background as a factor for enrollment into the study. Consequently, this may have introduced variations (i.e. diet and lifestyle differences) in the patient population, ultimately contributing to the polar results between the two studies.

Future research evaluating the effects of Cr supplementation should attempt to identify the subgroup of patients in which Cr therapy may be beneficial (i.e. T2DM patients with Cr deficiency). Furthermore, a large sample size with adequate power and sufficient duration of therapy may be desired for reliable and trustworthy results. Finally, future research may consider properly controlling for concomitant antidiabetic medications to truly isolate the effect size of Cr supplementation in patients with T2DM.

Although it has been suggested that Cr supplementation promotes glucose control, there is insufficient evidence in the literature to make a definitive conclusion about the effects of Cr in patients with T2DM. Current trials evaluating Cr on glycemic control has led to conflicting results. Results from a 3 month randomized single-blinded study evaluating adults with T2DM (35–67 years old) suggested that Cr supplementation (42 mcg/daily) significantly reduced both A1C (9.51 ± 0.26% to 6.68 ± 0.28%; p < 0.001) and FPG (197.65 ± 8.68 mg/dL to 103.68 ± 6.64 mg/dL; p < 0.001) [14]. However, results from a double-blinded RCT evaluating Cr as adjuvant therapy in patients with T2DM reported no statistically significant differences between the intervention (Cr 400 mcg/daily) and placebo group for A1C after 6 month [16]. Moreover, results from a double-blinded, placebo-controlled cross-over trial evaluating 30 patients with T2DM also reported no difference between control and Cr treated (200 mcg/daily) subjects in glucose control (A1C, FPG) [29].

In conclusion, Cr supplementation with brewer’s yeast may provide marginal benefits in lowering FPG in patients with T2DM compared to placebo control however Cr supplement as other formulations did not have any effect on A1C or FPG. Consequently, it would be premature to recommend Cr supplementation as part of the standard of care in patients with T2DM. Additional research with large sample sizes and well-designed RCTs are required to further understand which patient population would benefit the most from Cr supplement.

## Appendix

Search Strategy

PubMed

((“chromium” [Mesh] OR “chromium” [all fields]) AND (“diabetes mellitus, type 2” [Mesh] OR “ type 2 diabetes” [all fields] OR “type ii diabetes” [all fields] OR “t2dm” [all fields] OR “tiidm” [all fields] OR “NIDDM” [all fields] OR “non insulin dependent diabetes” [all fields] OR “type 2 dm” [all fields]) AND (“humans” [mesh]))

Cochrane library

((“chromium” OR “chromium”) AND (“diabetes mellitus, type 2” OR “ type 2 diabetes” OR “type ii diabetes” OR “t2dm” OR “tiidm” OR “NIDDM” OR “non insulin dependent diabetes” OR “type 2 dm”) AND (“humans”))

Embase

((‘chromium’/exp OR ‘chromium’:ti,ab) AND (‘diabetes mellitus, type 2’/exp OR ‘ type 2 diabetes’:ti,ab OR ‘type ii diabetes’:ti,ab OR ‘t2dm’ :ti,ab OR ‘tiidm’:ti,ab OR ‘NIDDM’:ti,ab OR ‘non insulin dependent diabetes’ :ti,ab OR ‘type 2 dm’:ti,ab) AND (‘humans’/exp))
